# A diagnosis of stroke-like migraine attacks after radiation therapy (SMART) as severe headache with stroke-like presentation

**Published:** 2016-10-07

**Authors:** Aileen O’Shea, Sinead Culleton, Hamed Asadi, Hong Kuan Kok, Alan O’Hare, John Thornton, Paul Brennan, Seamus Looby

**Affiliations:** 1 Neuroradiology and Neurointerventional Service, Department of Radiology, Beaumont Hospital, Dublin, Ireland; 2 Neuroradiology and Neurointerventional Service, Department of Radiology, Beaumont Hospital, Beaumont Road, Dublin, Ireland AND School of Health, Deakin University, Waurn Ponds, Australia

**Keywords:** Stroke-Like Migraine Attacks after Radiation Therapy (SMART), Headache, Cranial Irradiation

## Introduction

**Figure 1:**
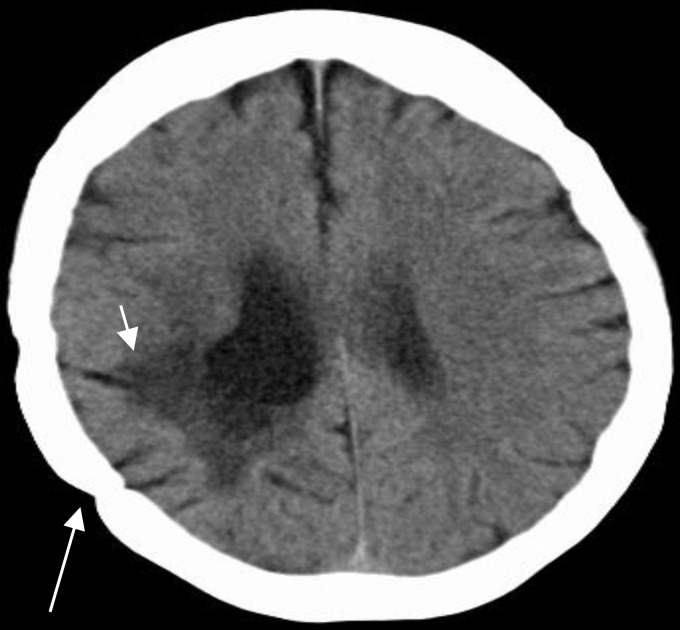
Noncontrast brain computed tomography (CT) demonstrating previous right parietotemporal craniotomy (long arrow) and the underlying encephalomalacia (short arrow)

A 52-year-old woman presented to the emergency department with gradual onset headache and expressive dysphasia. She had a left-sided facial droop and an unsteady gait on examination. An immediate non-contrast brain computed tomography (CT) was performed demonstrating evidence of prior right-sided neurosurgical intervention but no acute abnormality to account for her clinical findings (Figure 1). Further enquiry revealed a history of right parietal meningioma resection eleven years earlier followed by adjuvant cranial radiotherapy.

**Figure 2:**
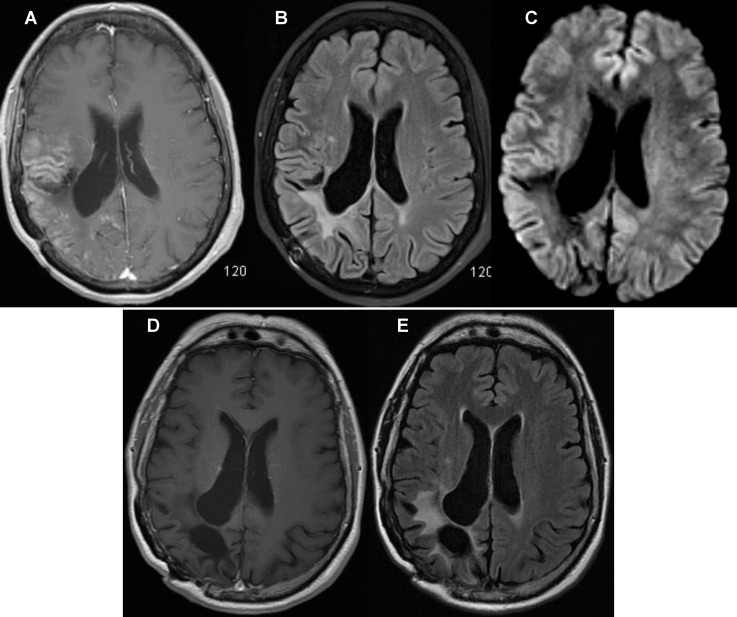
Brain magnetic resonance imaging (MRI): (A) Axial T1-weighted post-contrast, (B) Fluid-attenuated inversion recovery (FLAIR) and (C) Diffusion-weighted imaging showed gyriform enhancement in the right parietal and temporal lobes (thin arrow) with associated gyral swelling overlying the site of previous surgery (thick arrow). (D) T1-weighted post-contrast and (E) FLAIR repeat Imaging 6 month's later, demonstrated complete resolution of the above-mentioned findings.

A subsequent brain magnetic resonance imaging (MRI) demonstrated cortical thickening and a gyriform pattern of enhancement at the site of previous resection, with no convincing area of abnormal diffusion restriction (Figure 2, A-C). The patient’s symptoms gradually resolved over the course of few months and a repeat MRI brain revealed complete resolution of the previously demonstrated acute findings (Figure 2, D and E). 

In view of the clinical presentation of headaches and focal neurological deficits, these image findings in the setting of prior cranial irradiation is characteristic of stroke-like migraine attacks following radiation therapy, also known as SMART syndrome.

SMART syndrome was first described in 1995 and the exact incidence is unknown. It describes a late-onset adverse effect of cranial irradiation, following treatment for a cerebral neoplastic process, usually a malignancy.^1^ The clinical features of SMART syndrome are varied but usually consist of migrainous neuralgia with associated symptoms such as nausea and photophobia. Focal neurological deficits such as speech, visual and hearing disturbances, tremor, hemiparesis and hemiplegia have all been described, and seizures are noted in up to 82% of the patients.^2^

SMART syndrome generally shows a self-limiting course without persistent neurological effects, and imaging features resolve in accordance with abating symptomology,^3^ with 55% of patients recovered completely over approximately 2 months.^4^ However, interestingly, in a retrospective case series by Black et al., it was shown that up to 45% of patients may have persistent neurologic deficits, with approximately 27% of patients demonstrating permanent imaging abnormalities.^4^

Diagnostic investigations in SMART syndrome are centered on the exclusion of other possible etiologies, including tumor recurrence, acute stroke, posterior reversible encephalopathy syndrome (PRES), hemiplegic migraine and cerebral autosomal dominant arteriopathy with subcortical infarcts and leukoencephalopathy (CADASIL) syndrome.^2^ In tumor recurrence, enhancement close to the resection site is intransient and progressive, while in SMART, interval reimaging typically shows resolution of such findings. PRES tends to show bilateral imaging changes, and a family history is usually present in cases of autosomal dominant hemiplegic migraine or CADASIL, both of which can be confirmed by genetic testing. 

Unilateral increased T2-weighted signal in the cortex with gyral enhancement that resolves on subsequent imaging typically accompanied by resolution of symptoms distinguishes SMART from these other differential diagnoses.^1^ A detailed clinical history, electroencephalography (EEG) and cerebrospinal fluid analysis can help to exclude other entities. In those presenting with seizures, EEG analysis does not usually exhibit an epileptiform pattern,^5^ however this is not an absolute finding.^6^

SMART syndrome should be considered in patients previously treated with cranial irradiation who present with headaches and acute neurologic deficits, in conjunction with the characteristic MRI findings above. These typical and usually transient radiological abnormalities help to establish a diagnosis of SMART syndrome, and awareness of this late complication of cranial irradiation can avoid further unnecessary workup.

## Notes:

**How to cite this article:** O’Shea A, Culleton S, Asadi H, Kok HK, O’Hare A, Thornton J, et al. A diagnosis of stroke-like migraine attacks after radiation therapy (SMART) as severe headache with stroke-like presentation. Iran J Neurol 2016; 15(4): 237-9.
